# Appropriate Surgical Margins for Excision of Squamous Cell Carcinoma of the Lower Lip

**DOI:** 10.1055/a-2095-6885

**Published:** 2023-08-02

**Authors:** Jung Hyun Hong, Chan Woo Jung, Hoon Soo Kim, Yong Chan Bae

**Affiliations:** 1 Department of Plastic and Reconstructive Surgery, Pusan National University, School of Medicine, Busan, Korea; 2 Department of Dermatology, Pusan National University Hospital, Busan, Korea; 3 Biomedical Research Institute, Pusan National University Hospital, Busan, Korea

**Keywords:** margins of excision, lip cancer, squamous cell carcinoma

## Abstract

**Background** Squamous cell carcinoma (SCC) is the most common malignancy on the lower lip. Surgical excision, the standard treatment for SCC, requires full-thickness excision. However, no consensus exists about the appropriate surgical margin. Therefore, we investigated the appropriate surgical margin and excision technique by analyzing 23 years of surgical experience with lower-lip SCC.

**Methods** We reviewed 44 patients with lower-lip SCC who underwent surgery from November 1997 to October 2020. Frozen biopsy was performed with an appropriate margin on the left and right sides of the lesion, and the margin below the lesion was the skin above the sulcus boundary. If the frozen biopsy result was positive, an additional session was performed to secure a negative margin. Full-thickness excision was performed until the final negative margin. In each patient, the total number of sessions performed, final surgical margin, and recurrence were analyzed.

**Results** Forty-one cases ended in the first session, 2 ended in the second session, and 1 ended in the third session. The final surgical margins (left and right; *n* = 88) were 5 mm (66%), 7 mm (9%), 8 mm (2.3%), 10 mm (20.4%), and 15 mm (2.3%). During an average follow-up of 67.4 months (range, 12–227 months), recurrence occurred in one patient.

**Conclusion** The final surgical margin was 5 mm in 66% (58/88) of the cases, and 97.7% (86/88) were within 10 mm. Therefore, we set the first frozen biopsy margin to 5 mm, and we suggest that a 5-mm additional excision is appropriate when frozen biopsy results are positive.

## Introduction

Squamous cell carcinoma is the most common malignant tumor on the lower lip. As age increases, the risk of this condition rises, with the highest incidence in 70-to 80-year olds. Sun exposure, tobacco use, and genetic causes act in a multifactorial way to promote the development of squamous cell carcinoma, although its exact cause has not been identified. It is thought to be more prevalent in certain occupations, such as farmers and fishermen, but that is not necessarily the case, and the role of occupation as a risk factor remains a matter of debate. In general, it shows a male predominance, and most cases of lip cancer in the United States occur in men. There is also an inverse correlation between latitude and the incidence of squamous cell carcinoma, suggesting that ultraviolet radiation contributes to the occurrence of squamous cell carcinoma. Early detection of squamous cell carcinoma is often possible because it occurs in a position where it is easy to see. Therefore, its prognosis is generally favorable, with a 5-year survival rate exceeding 90% according to various studies. 1 2 3

The treatment methods for squamous cell carcinoma include surgery and radiotherapy. However, since radiotherapy takes a long time and the recurrence rate is high, surgical treatment is the most standard treatment method for patients without metastasis. 4 5

When wide excision is performed, a gross inspection of the surgical field cannot confirm whether the lesion has been completely excised. Furthermore, wide excision involves the removal of a substantial amount of normal tissue. Reconstruction should also consider the burden on the patient in terms of finances and time, and extensive reconstruction significantly reduces patients' functional and cosmetic satisfaction after surgery.

Mohs micrographic surgery (MMS) is a useful method for ensuring complete cancer removal, but it is time-consuming and costly. The authors have performed surgery on other parts of the face using the conventional MMS method. However, for tumors on the eyelid and lip, it is often difficult to select an appropriate reconstruction method, so MMS is not performed.

At many centers, the surgical method varies depending on the surgeon. When frozen biopsy is possible for patients with stage I/II cancer, some cases have been reported where frozen biopsies were performed with a 3-mm margin, but this has only been described in a single study. 1 Researchers have recommended securing a margin of at least 6 mm, and some surgeons prefer a surgical margin of at least 10 mm. 6 7 8 9 10 11 12 13

Therefore, we performed the first frozen biopsy at an appropriate distance from the gross boundary of the lesion. If negative, the remaining cancer tissue was removed, and if positive, frozen biopsy was performed again at an appropriate distance after additional resection. This proceeded until the negative frozen biopsy results were reported. In this workflow, the distance of the first frozen biopsy from the visual boundary and the location of the next frozen biopsy in case of positive frozen biopsy results greatly affected the operation time and results.

Accordingly, we analyzed patients with squamous cell carcinoma of the lower lip for 23 years (from 1997–2020). We investigated where the initial frozen biopsy was performed, at what interval, and the width of the final surgical margin. The aim of this study was (1) to evaluate the appropriate initial margin and additional intervals and (2) to establish a reference value of the surgical margin and length through a long-term follow-up study. We expect that the results of this study will be helpful in the treatment of squamous cell carcinoma of the lower lip in the future.

## Methods

### Patients

We retrospectively studied 44 patients treated from November 1997–October 2020 for squamous cell carcinoma on the lower lip. We performed surgery with frozen biopsy to determine the excision margin. The average age of the patients was 69.09 years (range, 43–90 years), and there were 24 men and 20 women.

### Surgical Method

The gross margin was marked based on palpation of the lesion, and the first surgical margin was flexibly designed to be 5–10 mm left and right from the gross margin, considering the tumor location and size. On the lower side, the surgical margin was the skin above the sulcus boundary.

**Fig. 1:**
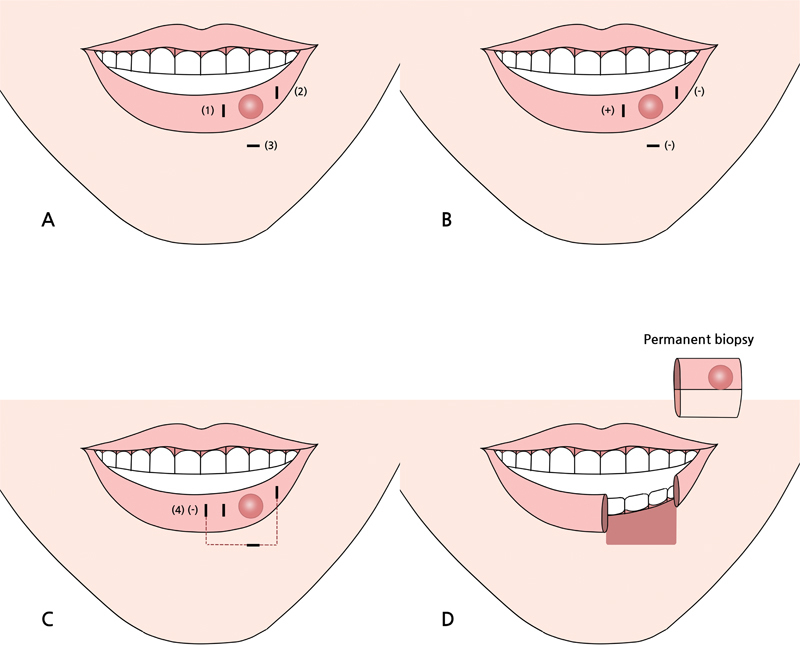
Surgical method. ( **A** ) The initial frozen biopsy is done at the right margin (1), the left margin (2), and the lower margin (3). ( **B** ) The biopsy result is positive at the right margin (1) and negative at the left margin (2) and lower margin (3). ( **C** ) An additional frozen biopsy is done 5-mm lateral from the initial right margin (4). ( **D** ) The biopsy result is negative in (4). Then, full layer excision is done and the excised mass is sent for permanent fixation and a definitive histopathologic examination.

Frozen biopsy (frozen section) was performed at the left, right, and lower margins. When the frozen biopsy result was positive, an additional frozen biopsy was performed 5 mm away from where the initial frozen biopsy was performed. This process continued until negative results on frozen biopsy were reported. After securing a negative margin, full-layer excision was performed along the surgical margin. The excised tissue was subjected to permanent fixation and a definitive histopathologic examination, and the defect was reconstructed ( Fig. 1 ).

### Study Method

For each patient, the total number of sessions, final surgical margin, and recurrence were investigated. One patient died of hematologic cancer unrelated to squamous cell carcinoma 2 months after surgery. Except for this patient, the average follow-up was 67.4 months (range, 12–227 months).

## Results

All 44 patients had negative results of the inferior frozen biopsy. Among the 88 initial left and right margins, the initial frozen margin was 5 mm in 60 margins (68.2%), 7 mm in 8 margins (9%), 8 mm in 2 margins (2.3%), and 10 mm in 18 margins (20.5%; Table 1 ).

**Table 1:** Initial frozen margins

Initial frozen margin (mm)	Number of margins (%)
5	60 (68.2)
7	8 (9.0)
8	2 (2.3)
10	18 (20.5)

**Fig. 2:**
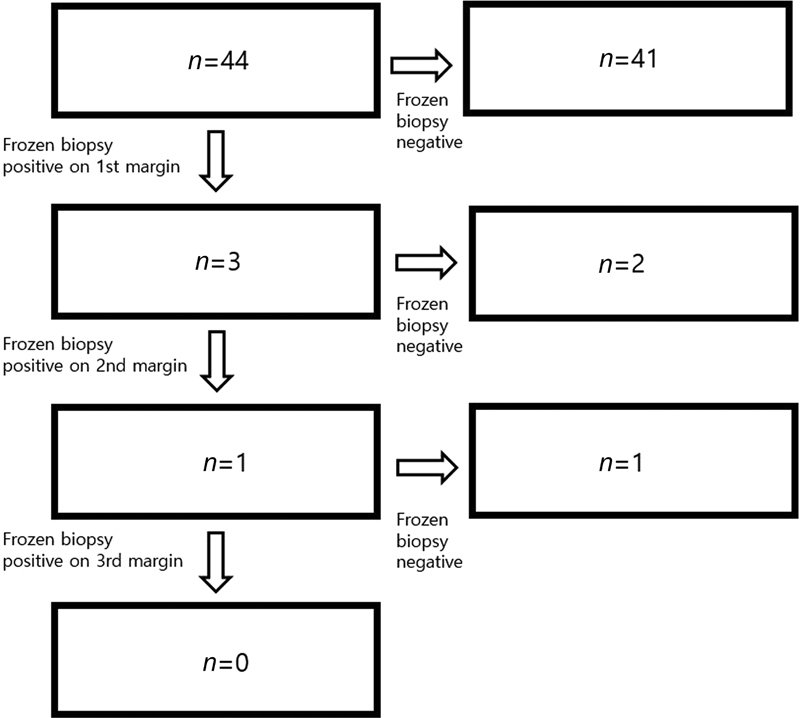
Arrow diagram for the frozen biopsies. In total, 44 patients underwent frozen biopsy for the first safety margin. Three patients had positive findings on the first frozen biopsy. In those patients, the first safety margin was 10, 5, and 5 mm, respectively. An additional frozen biopsy was done 5 mm lateral from the initial margin. Two patients had negative results from the second frozen biopsy result, and one patient (with a first safety margin of 5 mm) had positive results. An additional frozen biopsy was performed 5-mm lateral from the second margin. Finally, the third frozen biopsy was negative.

In total, 44 patients underwent frozen biopsy for the first safety margin. Three patients had positive findings on the first frozen biopsy. In those patients, the first safety margin was 10, 5, and 5 mm, respectively. An additional frozen biopsy was done 5-mm lateral from the initial margin. Two patients had negative results from the second frozen biopsy, while one patient (with a first safety margin of 5 mm) had positive results. An additional frozen biopsy was performed 5 mm lateral from the second margin. Finally, the third frozen biopsy was negative. Thus, 41 cases ended at the first session, two cases ended at the second session, and one case ended at the third session ( Fig. 2 ). The final surgical margin was 5 mm (58 margins, 66%), 7 mm (8 margins, 9%), 8 mm (2 margins, 2.3%), 10 mm (18 margins, 20.4%), and 15 mm (2 margins, 2.3%; Table 2 ). There were no positive margin results in the final pathology findings.

**Table 2:** Final surgical margins

Final surgical margin (mm)	Number of margins (%)
5	58 (66.0)
7	8 (9.0)
8	2 (2.3)
10	18 (20.4)
15	2 (2.3)

In an average follow-up of 67.4 months (range, 12–227 months), there was only one case of recurrence.

All patients received physical examinations, including palpation, during the follow-up period. Follow-up computed tomography (CT) was routinely performed at 6 months, 1 year, and 2 years after surgery to determine whether the disease had recurred. Follow-up was performed twice a week until the second week, then every month until 6 months, at 3-month intervals for up to 1 year, and then at 6-month intervals for up to 5 years.

One patient experienced recurrence. This patient refused surgery under general anesthesia due to the patient's general condition. Therefore, at the time of the first operation, metastasis to the neck lymph node was suspected, but neck dissection was not performed. After 20 months of follow-up, local recurrence was observed, but additional surgery was rejected.

Two patients simultaneously underwent radiation therapy after neck dissection at the otolaryngology department. These two patients both had T1N2bM0 (stage 4a) disease, and modified neck dissection was performed. In total, 60 Gy and 54 Gy of radiation therapy was administered to each of these patients, respectively, in 30 sessions over a 5-week period.

### Case 1

**Fig. 3:**
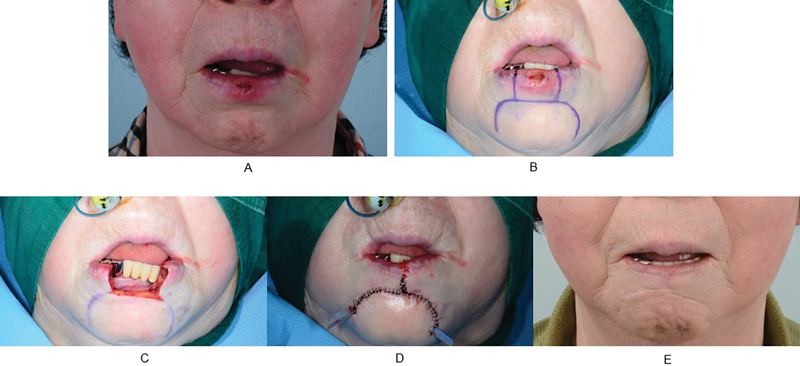
A case where frozen biopsy ended in the first session. ( **A** ) A preoperative photograph. ( **B** ) The preoperative design. ( **C** ) An intraoperative photograph after excision. ( **D** ) An immediate postoperative photograph. ( **E** ) A photograph at follow-up at postoperative 58 months.

A 70-year-old was diagnosed with squamous cell carcinoma on the lower lip. The patient had no specific medical history and the patient had noticed the mass 8 months before surgery. The size of the tumor was 9 × 7 mm, and it was located in the center of the lower lip. Facial, neck, and chest CT scans were taken, and no metastasis was observed. The stage was T1N0M0. The margins were all negative in the first session (left 5 mm, right 5 mm). Wide excision was performed, followed by reconstruction using a double barrel-shaped excision and local advancement flap. The follow-up period was 58 months. No recurrence or metastasis was found, and there were no complications following reconstruction. The patient was also satisfied with the postoperative scar in terms of cosmesis ( Fig. 3 ).

### Case 2

**Fig. 4:**
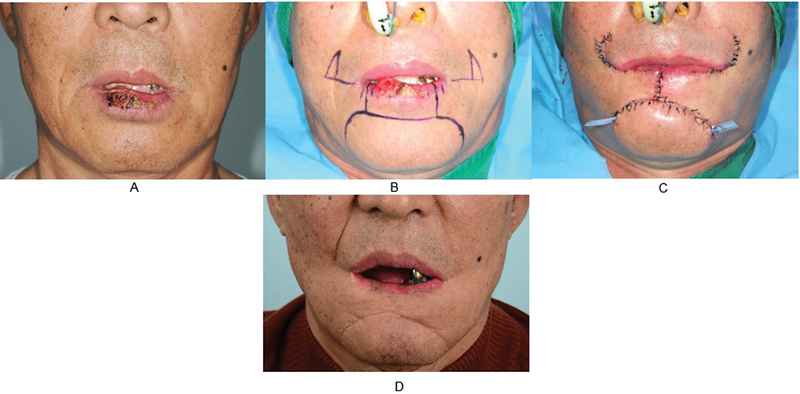
A case where frozen biopsy ended in the second session. ( **A** ) A preoperative photograph. ( **B** ) The preoperative design. ( **C** ) An immediate postoperative photograph. ( **D** ) A photograph at follow-up at postoperative 60 months.

A 63-year-old was diagnosed with squamous cell carcinoma on the lower lip. The patient had a history of hypertension and cerebral hemorrhage, and 3 years before surgery, laser removal was performed once at a local clinic. However, the tumor recurred thereafter. The size of the tumor was 16 mm × 16 mm, and it was located in the center and right side of the lower lip. Facial, neck, and chest CT scans were taken, and no metastasis was observed. The stage was T1N0M0. The margin was positive in the first session (right: 5 mm). The margin was negative in the second session (right 5 mm). Wide excision was performed, followed by reconstruction using the bilateral Webster modification of the Bernard technique. No recurrence or metastasis was found, and there were no complications following reconstruction. The patient was also satisfied with the postoperative scar in terms of cosmesis ( Fig. 4 ).

### Case 3

**Fig. 5:**
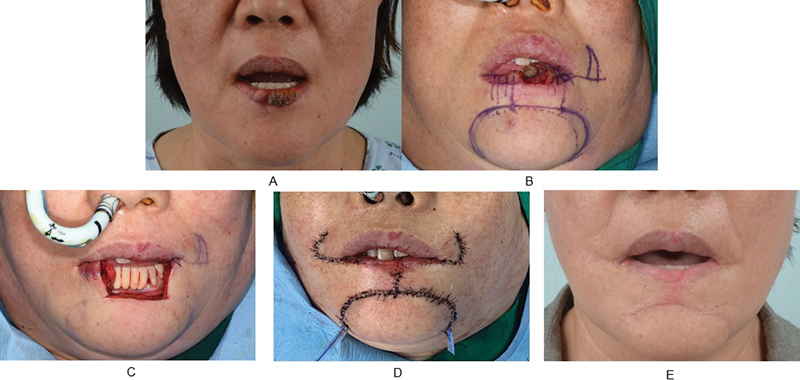
A case where frozen biopsy ended in the third session. ( **A** ) A preoperative photograph. ( **B** ) The preoperative design. ( **C** ) An intraoperative photograph after excision. ( **D** ) An immediate postoperative photograph. ( **E** ) A photograph at follow-up at postoperative 15 months.

A 59-year-old was diagnosed with squamous cell carcinoma on the lower lip. The patient had a history of hypertension. The size of the tumor was 15 × 12 mm, and it was located in the center and left side of the lower lip. Facial, neck, and chest CT scans were taken, and no metastasis was observed. The stage was T1N0M0. The margin was positive in the first and second sessions (right 5 mm). The margin was negative in the third session (right 5 mm). Wide excision was performed, followed by reconstruction using the bilateral Webster modification of the Bernard technique. No recurrence or metastasis was found, and there were no complications following reconstruction. The patient was also satisfied with the postoperative scar in terms of cosmesis ( Fig. 5 ).

## Discussion

Squamous cell carcinoma is the most common malignant tumor on the lower lip. It occurs more frequently in men than in women, and the average age of diagnosis is 66.1 years. 3 14 15

For complete excision of cancer cells, as described above, we performed frozen biopsy on the left and right from the gross margin, considering the tumor location and size. On the lower side, the margin was the skin above the sulcus boundary. After securing additional margins at intervals of 5 mm until negative results were obtained, full-thickness excision was performed according to the final surgical margin.

In this retrospective study of 44 patients, the final surgical margin was 5 mm in 66% (58/88) of margins, and 86 (97.7%) were 10 mm or less. During an average of 67.4 months of follow-up (range, 12–227 months), only one patient experienced recurrence (1/44; 2.3%). Based on these results, we recommend setting the first frozen biopsy margin at 5 mm, and if needed, additional biopsies can be performed at intervals of 5 mm.

Previous studies recommended excising 10 mm of normal-looking tissue beyond the border of the tumor as a safe margin. 6 7 8 9 10 11 12 Another study suggested a margin of at least 6 mm. 13 However, surgery involving the excision of such a large amount of normal tissue, coupled with the subsequent reconstruction, is financially burdensome and time-consuming for patients, and their functional and cosmetic satisfaction after surgery may be significantly reduced.

A previous study reported that 3 mm was suitable for the first surgical margin in early-stage cancers (stage I/II). However, no additional studies have investigated the feasibility of a 3-mm margin. This recommendation is also limited to patients with early-stage disease, 1 underscoring the importance of research on a broader patient population. Moreover, metastasis easily occurs from squamous cell carcinoma on the lower lip to the lymph node of the neck, especially if the maximal thickness of the tumor is more than 6 mm. 16 In this study, metastasis was determined by biopsy when an enlarged lymph node in the neck was observed on a CT scan. The authors surgically removed the tumors in all patients. Two patients with neck metastases also underwent neck dissection surgery and radiotherapy at the otolaryngology department.

This study has several limitations. First, patients who underwent surgery recently had a relatively short follow-up period, which was sometimes shorter than the average recurrence time (67.4 months). Additional follow-up will be required in the future. Second, in the early period of the study, the first frozen margin was determined according to the operator's experiential competency, and in some cases, it was more than 5 mm.

To summarize, we performed surgery in 44 patients diagnosed with squamous cell carcinoma on the lower lip over a 23-year period from 1997–2020. Only one patient had recurrence during follow-up. Of the 88 left and right final surgical margins, 86 (97.7%) were within 10 mm, and 58 (66.0%) were within 5 mm. Only three cases lasted beyond the second session.

Therefore, the method described herein is efficient in terms of time and cost, and it is safe from the risk of recurrence. Based on these findings, 5 mm is considered appropriate as the first frozen biopsy margin. We expect that these findings will be helpful for the surgical treatment of squamous cell carcinoma on the lower lip.
